# Efficacy of High-Flow Nasal Cannula versus Conventional Oxygen Therapy in Obese Patients during the Perioperative Period: A Systematic Review and Meta-Analysis

**DOI:** 10.1155/2022/4415313

**Published:** 2022-09-20

**Authors:** Rong Zhou, Hao-Tian Wang, Wei Gu

**Affiliations:** ^1^Department of Anesthesiology, Nanjing Drum Tower Hospital, The Affiliated Hospital of Nanjing University Medical School, 321 Zhongshan Road, Nanjing 210008, Jiangsu, China; ^2^Queen Marry School of Nanchang University, 461 Bayi Road, Nanchang 330006, China

## Abstract

**Background:**

Obesity is a risk factor for severe airway obstruction and hypoxemia. High-flow nasal cannula (HFNC) is considered as a novel method for oxygen therapy, but the efficacy of HFNC for obese patients is controversial. This meta-analysis aimed to assess the efficacy of HFNC compared with conventional oxygen therapy (COT) in obese patients during the perioperative period.

**Methods:**

We searched the PubMed, Embase, Web of Science, the Cochrane Library, and Google scholar databases for randomized controlled trials (RCTs) that compared the efficacy of HFNC with COT in obese patients during the perioperative period. The primary outcome was the incidence of hypoxemia, while the secondary outcomes included the lowest SpO_2_, the need for additional respiratory support, and the hospital length of stay (LOS).

**Results:**

Twelve trials with 798 obese patients during the perioperative period were included. Compared with COT, HFNC reduced the incidence of hypoxemia (RR, 0.60; 95% CI, 0.43 to 0.83; *P*=0.002; *I*^2^ = 24%; 8 RCTs; *n* = 458), increased the lowest SpO_2_ (MD, 2.88; 95% CI, 1.53 to 4.22; *P* < 0.0001; *I*^2^ = 32%; 5 RCTs; *n* = 264), decreased the need for additional respiratory support (RR, 0.43; 95% CI, 0.21 to 0.88; *P*=0.02; *I*^*2*^ = 0%; 3 RCTs; *n* = 305), and shortened the hospital LOS (MD, −0.31; 95% CI, −0.57 to −0.04; *P*=0.02; *I*^2^ = 0%; 3 RCTs; *n* = 214).

**Conclusions:**

This meta-analysis showed that compared with COT, the use of HFNC was able to reduce the incidence of hypoxemia, increase the lowest SpO_2_, decrease the need for additional respiratory support, and shorten the hospital LOS in obese patients during the perioperative period. Well-organized trials with large sample size should be conducted to support our findings.

## 1. Introduction

Obesity is defined as excessive body fat tissue accumulation that confers risks for metabolic disorders. A person with a body mass index (BMI) ≥30 kg/m^2^ is considered as obese [1]. Obesity and obesity-related diseases are the risk factors for cardiovascular and respiratory diseases, resulting in the decrease of life quality and expectancy [2]. Obese patients have a higher risk of difficult mask ventilation, and difficult tracheal intubation compared with the nonobese [3]. The compliance of respiratory organs, lung volumes, and a reduced functional residual capacity is decreased in obese patients [4]. Besides, obese patients are afflicted by obstructive sleep apnea [4]. These situations were exacerbated during the perioperative period and elevated risk of hypoxemia in obese patients. Therefore, appropriate oxygen therapy is crucial to the prevention of perioperative complications in obese patients. The conventional oxygen therapy (COT) is provided by nasal cannulas, or facemasks with limited flow rate (≤15 L/min). It has limited ability to meet the inspiratory demands of the obese patients with high risk of hypoxemia [5]. High-flow nasal cannula (HFNC), as a new modality oxygen therapy, is capable of delivering a high flow rate (≥20 L/min) of heated, humidified gas at an adjustable concentration without recourse to invasive or noninvasive ventilation [6]. The American clinical guideline suggested that compared to COT, HFNC as postextubation management may reduce the reintubation rates [7]. The application of HFNC is recommended in patients with hypoxemic respiratory failure. Likewise, the use of HFNC is conditionally recommended in obese patients after cardiac or thoracic surgery [8]. A recent meta-analysis investigated the efficacy of HFNC in comparison to COT or noninvasive ventilation (NIV) in obese patients in the peri- and postprocedures. The results demonstrated that the HFNC could prolong the safe apnea time, without any benefit on the reduction of hypoxemia and CO_2_ elimination [7]. At present, it remains unclear whether HFNC is superior to COT in obese patients in reducing hypoxemia or enhancing oxygenation. To explore the advantages of HFNC in obese patients, our meta-analysis aimed at comparing the incidence of hypoxemia, the lowest SpO_2_, the need for additional respiratory support, and the hospital length of stay (LOS) between obese patients receiving HFNC and those using COT.

## 2. Materials and Methods

### 2.1. Protocol and Registration

The meta-analysis was conducted in accordance with the recommendation of Preferred Reporting Items for Systematic Reviews and Meta-analyses (PRISMA) statement [9], and registered the review protocol on INPLASY PROTOCOL (INPLASY 2021110106).

### 2.2. Literature Search

We searched the PubMed, Embase, Web of Science, the Cochrane Library, and Google scholar databases from inception to August 10, 2022, using the following keywords: (“HFNC” OR “HFNO” OR “NHF” OR “high flow nasal cannula” OR “high flow nasal therapy” OR “high flow nasal oxygen” OR “nasal high flow”) AND (“obesity” OR “obes^*∗*^” OR “bariatric” OR “fat” OR “corpulent”) AND (“trial”) limited to randomized controlled trials (RCTs). No restriction was imposed on language, sample size, gender, and study location. Detailed search strategies were demonstrated in Appendix 1. Besides, we reviewed the reference lists of retrieved trials for identifying additional trials, and manually searched the relevant articles by Google scholar. We included gay literature to reduce the potential publication bias by performing additional searches for conference proceeding in Web of Science (Core Collection), and registered trials in https://ClinicalTrials.gov and ChiCTR (https://www.clinicaltrials.gov, https://www.chictr.org.cn). EndNote X9 was used for managing the searched literature.

### 2.3. Inclusion Criteria and Exclusion Criteria

#### 2.3.1. Inclusion Criteria

The eligibility criteria for included trials are listed below by population, intervention, comparator, outcomes, and study characteristics, according to the PICOS (Population, Intervention, Comparison, Outcomes, Study design) strategy: (a) Population: adult patients (age ≥18 years) with obesity (BMI ≥30) during the perioperative period; (b) Intervention: the application of HFNC; (c) Comparison: the use of COT [e.g., facemask or low flow nasal oxygenation]; (d) Outcomes: inclusion of at least one of the predefined outcomes: incidence of hypoxemia, the lowest SpO_2_, the need for additional respiratory support, and the hospital LOS. (e) Study design: RCTs.

#### 2.3.2. Exclusion Criteria

(1) Those published as protocols, review articles, abstracts, editorials, and letters; (2) those presented as a crossover, retrospective, observational, cohort, or case-control study other than original research; (3) those ongoing or unpublished grey literature.

### 2.4. Outcomes and Definition

The primary outcome was the incidence of hypoxemia, while the secondary outcomes included the lowest SpO_2_, the need for additional respiratory support, and the hospital LOS. The definition for hypoxemia was varied in included trials, such as SpO_2_ < 90%, SpO_2_ < 92%, and SpO_2_ < 95%. The additional respiratory support was defined as an escalation in oxygen support therapy, including the intermittent positive-pressure ventilation, continuous positive airway pressure (CPAP), NIV, HFNC, or reintubation.

### 2.5. Selection Criteria and Date Extraction

#### 2.5.1. Selection Criteria

Two authors (R. Z. and H. T. W.) examined the titles and abstracts of the retrieved trials independently, and reviewed the full texts of the potentially eligible trials based on the inclusion and exclusion criteria. Any disagreement was resolved by a third author (W. G.).

#### 2.5.2. Date Extraction

Two authors independently extracted the trial characteristics, which were summarized in a standardized Excel file. The following information were retrieved from each trial: first author, year of publication, location, population, clinical setting, sample size, interventional time point, intervention and control details, the incidence of hypoxemia, the lowest SpO_2_, the need for additional respiratory support, and the hospital LOS. Disagreements were adjudicated by a third author.

### 2.6. Risk of Bias Assessment

The methodological quality of each trial was assessed by two authors through the Cochrane Risk of Bias Tool [10], and the risk of bias of each trial was described as “low,” “high,” or “unclear” [11]. The following domains were considered: random sequence generation (selection bias), allocation concealment (selection bias), blinding of participants and personnel (performance bias), blinding of outcome assessment (detection bias), incomplete outcome data (attrition bias), selective reporting (reporting bias), and other bias. We categorized the trials with low risk of bias for all domains as being at low risk of bias, the trials only owning one high bias as being at high risk, and all other trials were considered as unclear. Disagreements were settled through discussion.

### 2.7. Statistical Analysis

Dichotomous outcomes were presented as the relative risks (RRs), and continuous outcomes were presented as the mean differences (MDs), both with corresponding 95% confidence intervals (CIs). For continuous outcomes presented as the median (25th and 75th percentile), we converted inter-quartile ranges to standard deviation using formula conversion suggested by the Cochrane Collaboration [11]. Statistical heterogeneity was quantified by the *I*^2^tatistica, and the *I*^2^ ver 50% indicated significant heterogeneity. The intention-to-treat principle was used for performing the analyses. We adopted a priori random-effects model to pool the outcome data, on the assumption of heterogeneity across the included trials. Differences of the outcomes were graphically displayed with a forest plot, and a *P* value of <0.05 was considered to be statistically significant. To assess the potential impact of the findings from a single trial on the overall meta-analytical outcome, we adopted sensitivity analysis with a leave-one-out approach. The statistical analyses were performed using Review Manager, Version 5.3.

## 3. Results

### 3.1. Trial Selection

**Figure 1:**
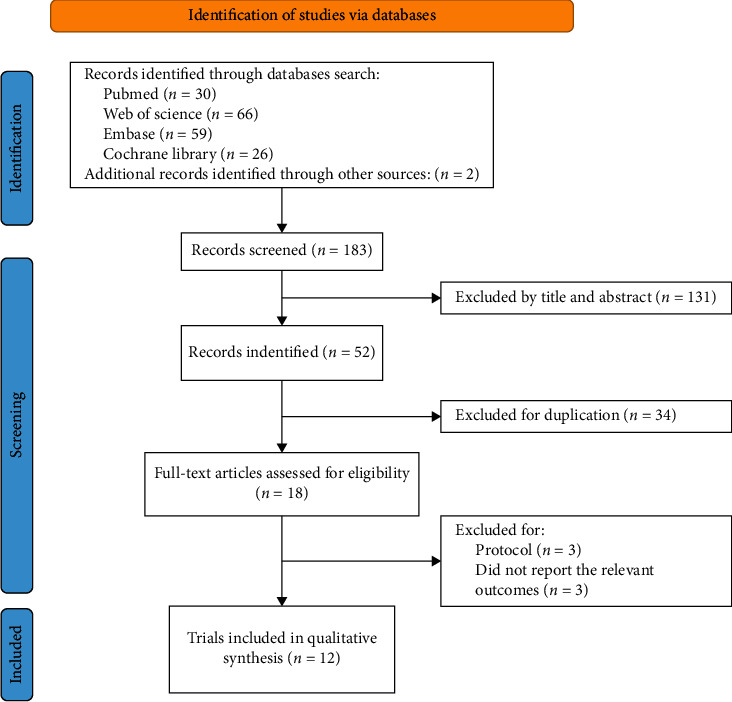
PRISMA flow diagram.

The process of trial selection is shown in Figure 1. First, the initial search yielded 183 records from the databases, and 131 records were excluded based on titles and abstracts. Of these records, 34 duplicates were excluded, and 18 records were thought to be potentially eligible. Second, after reviewing full texts in accordance with the inclusion criteria, 3 records were trial protocols, and 3 records did not include our outcomes. Finally, 12 trials were included in our meta-analysis [12–23].

### 3.2. Trial Characteristics

The basic characteristics of the included trials are summarized in Table 1. These included 12 trials were published from 2015 to 2022, and the population sizes ranged from 40 to 155, with a total of 798 patients. Our meta-analysis included the cardiothoracic surgery [12, 13], bariatric surgery [14, 16, 18–23], colonoscopy [15], and elective surgery [17]. The interventional time point was varied among included trials. Of the 12 trials, 5 assessed the beneficial effect of HFNC during the postoperative period [12–14, 18, 21], while 6 trials were conducted during anesthesia induction [16, 17, 19, 20, 22, 23], 1 trial was performed at the sedation of colonoscopy [15]. The oxygen flow of HFNC ranged from 25 to 120 L/min, and in the COT group, the oxygen was delivered through facemask or nasal cannula (2∼15 L/min). Eight trials provided the incidence of hypoxemia [14, 15, 18–23]. While three trials used the definition of SpO_2_ < 90% [15, 19, 21], four trials used the definition of SpO_2_ < 92% [14, 18, 22, 23], and one trial used the definition SpO_2_ < 95% [20].

### 3.3. Risk of Bias Assessment

**Figure 2:**
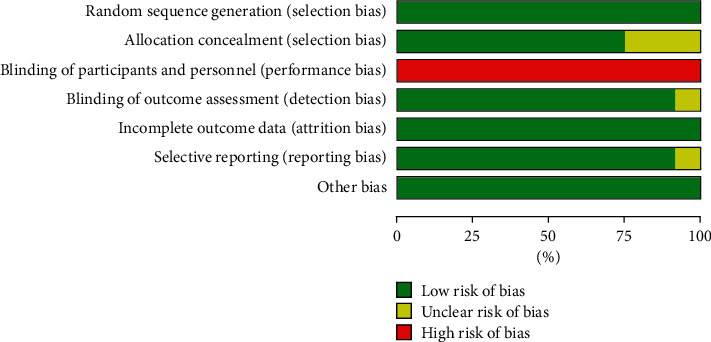
Risk of bias for each trial.

**Figure 3:**
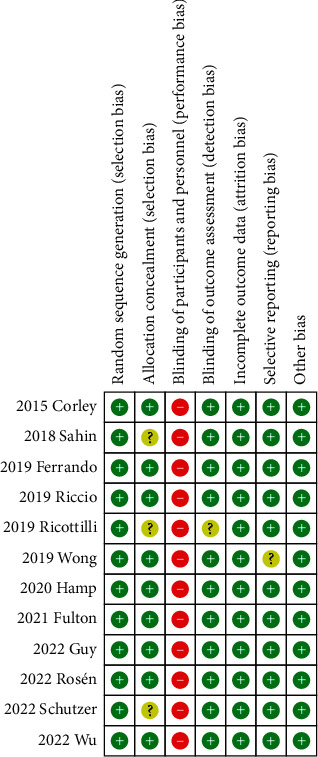
Summary of risk of bias.

The risks of bias of individual trials are summarized in Figures 2 and 3. Considering the impossibility of blinding among patients and medical staff, performance bias was high in all studies. In addition to the performance bias, eight trials were categorized as being at low risk of bias [12, 14, 15, 18–21, 23], and four trials as being unclear [13, 16, 17, 22].

### 3.4. Outcomes

#### 3.4.1. Primary Outcome

**Figure 4:**
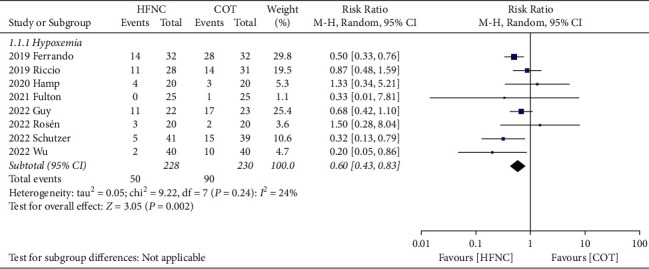
Forest plot comparing the incidence of hypoxemia between HFNC and COT.

Incidence of hypoxemia. Eight trials involving a total of 458 patients (HFNC group, *n* = 228 vs. COT group, *n* = 230) provided data on the incidence of hypoxemia [14, 15, 18–23]. The incidence of hypoxemia was 21.93% and 39.13% in the HFNC and COT group, respectively. Our meta-analysis revealed that the incidence of hypoxemia was lower in the HFNC compared to the COT group (RR, 0.60; 95% CI, 0.43 to 0.83; *P*=0.002; *I*^2^ = 24%; 8 RCTs; *n* = 458; Figure 4). Sensitivity analysis also demonstrated no significant influence on the incidence of hypoxemia by omitting certain trials. We did not analyze the publication bias because only eight trials were available.

#### 3.4.2. Secondary Outcomes

**Figure 5:**
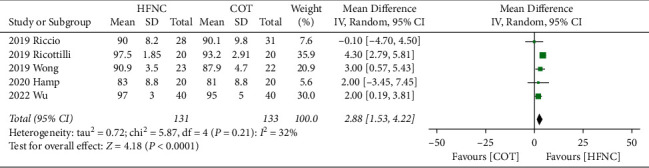
Forest plot comparing the lowest SpO_2_ between HFNC and COT.

**Figure 6:**
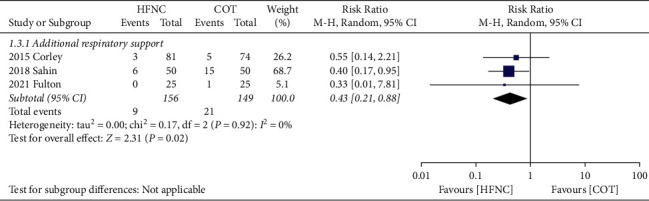
Forest plot comparing the need for additional respiratory support between HFNC and COT.

**Figure 7:**
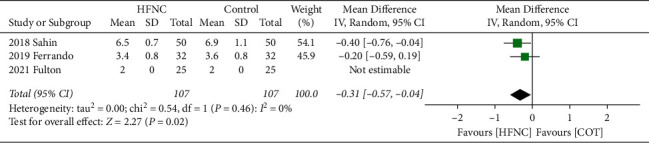
Forest plot comparing hospital LOS between HFNC and COT.

The lowest SpO_2_, the need for additional respiratory support, and the hospital LOS. Our results demonstrated that the lowest SpO_2_ was significantly increased by HFNC compared to COT (MD, 2.88; 95% CI, 1.53 to 4.22; *P* < 0.0001; *I*^2^ = 32%; 5 RCTs; *n* = 264; Figure 5). Sensitivity analysis demonstrated consistent findings when the five trials were removed one at a time. The use of HFNC was associated with a decrease of additional respiratory support compared to COT (RR, 0.43; 95% CI, 0.21 to 0.88; *P*=0.02; *I*^*2*^ = 0%; 3 RCTs; *n* = 305; Figure 6). Sensitivity analysis demonstrated consistent findings when the three trials were removed one at a time. Furthermore, the HFNC was associated with the shorter hospital LOS compared to COT (MD, −0.31; 95% CI, −0.57 to −0.04; *P*=0.02; *I*^2^ = 0%; 3 RCTs; *n* = 214; Figure 7). Sensitivity analysis was not performed because only two trials were available for this outcome comparison.

## 4. Discussion

### 4.1. Main Findings

Our study is the first meta-analysis focusing on a comparison between HFNC and COT in obese patients during the perioperative period. The results showed that the risk of hypoxemia is decreased 60% in the HFNC compared to the COT group. The application of HFNC was associated with the increased level of lowest SpO_2_, the decrease of the need for additional respiratory support, and a reduced hospital LOS in comparison to COT.

### 4.2. Comparison with Previous Meta-analyses

Previous meta-analyses assessing the efficacy of HFNC in varied clinical scenarios have been published, including patients with acute respiratory failure, COPD, obesity, and patients with planned extubation in ICU [7, 24–27]. Of these studies, only two studies explored the application of HFNC in obese patients [7, 25]. Hung's study showed HFNC might prolong the safe apnea time, but did not improve oxygenation compared to COT or NIV in obese patients during the peri- and postprocedural period [7]. Another study involving 3 trials also showed that there was no significant advantages of improving oxygenation in HFNC compared to the COT group in obese patients who underwent cardiac surgery [25]. Nevertheless, due to limited trials being included in previous studies, the conclusion might be controversial in obese patients.

Spence's meta-analysis focusing on the surgical patients during the intraoperative period showed that HFNC could reduce the risk of hypoxemia, and improve oxygenation in the intraoperative setting compared to COT [28]. In concert with this finding, we found that in obese patients, the use of HFNC could reduce the incidence of hypoxemia, and increase the lowest SpO_2_. Previous studies showed that compared to COT, the use of HFNC in the postoperative period may decrease the escalation of respiratory support, especially for obese patients, but it was lack of evidence [5, 29–31]. Similarly, we found a reduction of additional respiratory therapy in obese patients receiving HFNC compared with COT. The potential mechanism is that the HFNC could enhance the mucociliary clearance, and decrease the risk of patient self-inflicted lung injury [32]. However, only three trials were included in our study, and further studies were required to confirm this finding. Besides, our study demonstrated that the HFNC could shorten the hospital LOS of obese patients, but Chaudhuri's meta-analysis did not find a difference on this outcome between HFNC and COT [31]. With respect to the hospital LOS, there were relatively small sample size and limited trials in our meta-analysis, further trials were warranted to confirm it.

### 4.3. Implications for Clinical Practice and Mechanism

The obese patients have a higher risk for pneumonia, atelectasis, and other postoperative complications [33, 34]. HFNC with high adherence rate, comfortable experience, and lower costs has emerged as a new oxygen supportive treatment, but the efficacy of HFNC for obese patients during the perioperative period is controversial. Our meta-analysis found for obese patients the use of HFNC could reduce the incidence of hypoxemia, increase level of lowest SpO_2_, decrease the need for additional respiratory support, and shorten the hospital LOS.

The mechanism might be associated with the following reasons: (1) The modest amount of positive end-expiratory pressure generated by HFNC could greatly flush potential nasopharyngeal dead space, reduce the carbon dioxide levels, as well as improve ventilation and perfusion matching for the obese patients [35]. (2) HFNC could ameliorate the clearance of respiratory secretions, and reduce the incidence of upper airway obstruction [27]. (3) HFNC could reduce the work of breathing, and optimize the inspiratory air-flow dynamics and oxygenation in the obese patients [36, 37].

### 4.4. Strengths and Limitations

The strengths of our study lie in the population included in all obese patients during the perioperative period. Second, we only focused on the efficacy of HFNC versus COT in the obese patients, and excluded NIV, CPAP, and other oxygen treatments. Third, we prioritized the patient-centered outcomes including hypoxemia, the lowest SpO_2_, additional respiratory support, and the hospital LOS rather than some physiologic outcomes. Finally, we used the intention-to-treat principle and a random-effects model for a more conservative estimate accounting for clinical heterogeneity.

Our study also has limitations. First, the patient characteristics (age, medical comorbidities, obesity degree, and ASA), and clinical characteristics (the definition of hypoxemia, interventional time point, and the type of procedure) all these might affect the pooled results. We did not perform the subgroup analysis, due to the limited trials. Second, because of few included trials, the conclusions for some outcomes may not have clinical significance. Third, formula conversion was used for the data that were not represented by the meanula [11], which might affect the stability of results.

## 5. Conclusion

In our meta-analysis, compared with COT, the use of HFNC was able to reduce the incidence of hypoxemia, increase the lowest SpO_2_, and shorten the hospital LOS in obese patients during the perioperative period. Therefore, HFNC may be superior to COT for reducing hypoxemia or enhancing oxygenation in obese patients during the perioperative period, but further large-scale and well-organized clinical trials are required to confirm the efficacy of HFNC in the obese patients. Although in our study outcomes did not demonstrate statistical heterogeneity, the definition of hypoxemia and the intervention time point were varied in included trials. Therefore, for the further research, it is important for to keep consistent in the intervention time point, obese degree, the surgery type, the definition of hypoxemia, as well as the exact threshold for additional respiratory support.

## Figures and Tables

**Table 1:** Basic characteristics of the included clinical trials.

Author, year, location	Population	Clinical setting	Number of patients (H/C)	Intervention time point	Intervention details	Control details	Outcomes
Corley [12], 2015, Australia	Patients aged ≥18, with a BMI ≥30	Cardiothoracic surgery	155 (81/74)	Postextubation	The gas flow rate was 35∼50 L/min.	The gas flow rate was 2∼4 L/min via nasal cannula or 6 L/min via facemask.	③
					FiO₂: NR.	FiO₂: NR.	

Sahin [13], 2018, Turkey	Patients with a BMI > 30	Coronary artery bypass grafting	100 (50/50)	Postextubation	The gas flow rate was 25∼40 L/min.	The gas flow rate was 2∼4 L/min via facemask.	③④
					FiO₂ = 50%.	FiO₂: NR	

.Ferrando [14], 2019, Spain	Patients with a BMI ≥ 35	Laparoscopic bariatric surgery	64 (32/32)	Postoperation	The gas flow rate was 60 L/min.	The gas flow rate was 15 L/min via facemask.	①④
					FiO₂ = 50%.	FiO₂ = 50%.	

Riccio [15], 2019, USA	Patients aged 18∼80, with a BMI > 40	Elective colonoscopy	59 (28/31)	5 min before and during the sedation period	The gas flow rate was 60 L/min.	The gas flow rate was 4 L/min via nasal cannula.	①②③
					FiO₂ = 36∼40%.	FiO₂ = 36∼40%.	

Wong [17], 2019, Canada	Patients aged ≥ 18, with a BMI ≥ 40	Elective surgery	45 (23/22)	Preoxygenation	The gas flow rate was 40 L/min.	The gas flow rate was 15 L/min via facemask.	②
					FiO₂ = 100%	FiO₂ = 100%.	

Ricottilli [16], 2019, Belgium	Obese patients, BMI: (40.6±3.79)	Bariatric surgery	40 (20/20)	Preoxygenation and duration of apnea	The gas flow rate was 50∼70 L/min.	The gas flow rate was 12 L/min via facemask.	②
					FiO₂ = 100%.	FiO₂ = 100%.	

Hamp [19], 2020, Austria	Adult patients with a BMI > 40	Bariatric surgery	40 (20/20)	Apneic oxygenation	The gas flow rate was 120 L/min.	The gas flow rate was 10 L/min via nasal cannula.	①②
					FiO₂ = 100%	FiO₂ = 100%.	

Fulton [18], 2021, Australia	Patients aged ≥ 18, with a BMI ≥30	Elective bariatric surgery	50 (25/25)	Postoperation	The gas flow rate was 50 L/min.	The gas flow rate was 2 L/min via nasal cannula.	①③④
					FiO₂ = 50%.	FiO₂ = 50%.	

Schutzer-Weissmann [22], 2021, UK	Patients aged 18∼65 years, with a BMI >40	Bariatric surgery	80 (41/39)	Preoxygenation and duration of apnea	The gas flow rate was 35∼70 L/min.	The gas flow rate was 15 L/min via facemask.	①
					FiO₂: NR.	FiO₂: NR.	

Guy [20], 2021, Australia	Patients aged ≥ 18, with a BMI ≥35	Bariatric surgery	45 (22/23)	Apneic period	The gas flow rate was 70 L/min.	The gas flow rate was 4 L/min via nasal cannula.	①
					FiO₂ = 100%.	FiO₂ = 100%.	

Rosén [21], 2022, Sweden	Patients aged 18∼60 years, with a BMI >35	Laparoscopic bariatric surgery	40 (20/20)	Postoperation	The gas flow rate was 40 L/min.	The gas flow rate was 2 L/min via nasal cannula.	①
					FiO₂ = 30%.	FiO₂ = 30%.	

Wu [23], 2022, Taiwan, China	Patients aged 20∼65, with a BMI>30	Laparoscopic sleeve gastrectomy	80 (40/40)	Preoxygenation	The gas flow rate was 30∼50 L/min.	The gas flow rate was 15 L/min via facemask.	①②
					FiO₂ = 100%	FiO₂ = 100%	

Outcomes: ① = Hypoxemia; ② = Minimum SpO₂; ③ = Additional respiratory support; ④ = Hospital LOS; FiO₂ = fraction of inspired; SpO₂ = peripheral oxygen saturation; NR = no record.

## Data Availability

The data used to support the findings of this study are included within the article.

## Glossary

BMI: Body mass index
HFNC: High-flow nasal cannula
COT: Conventional oxygen therapy
PRISMA: Preferred Reporting Items for Systematic Reviews and Meta-analyses
LOS: Length of stay
RCT: Randomized controlled trial
RR: Relative risk
MD: Mean difference
CI: Confidence interval
NIV: Non-invasive ventilation
CPAP: Continuous positive airway pressure.

## Additional Points

Question: Is high-flow nasal cannula (HFNC) better than conventional oxygen therapy (COT) in obese patients during the perioperative period? Findings: HFNC could reduce the incidence of hypoxemia, increase the lowest SpO_2_, reduce the need for additional respiratory support, and shorten the hospital length of stay (LOS) in obese patients during the perioperative period. Meaning: HFNC may be superior to COT for reducing hypoxemia or enhancing oxygenation in obese patients during the perioperative period.

## Supplementary Materials

Supplementary Materials Supplement 1: Appendix 1. Search strategy.
