# Supplementary material for: Efficacy of High-Flow Nasal Cannula versus Conventional Oxygen Therapy in Obese Patients during the Perioperative Period: A Systematic Review and Meta-Analysis
Source: Can Respir J. 2022 Sep 20;2022:4415313. doi: 10.1155/2022/4415313 (PMC9553645; doi:10.1155/2022/4415313)
Supplement: Supplementary Materials — Supplement 1: Appendix 1. Search strategy. [file 4415313.f1.doc]

**Appendix 1: Search strategy**

**Source: PubMed**

**Searched on:** August 10, 2022

**Results: 30**

| Search | Query | Results |
| --- | --- | --- |
| #1 | "HFNC"[Title/Abstract] | 990 |
| #2 | "HFNO"[Title/Abstract] | 134 |
| #3 | "NHF"[Title/Abstract] | 630 |
| #4 | "high flow nasal"[Title/Abstract] | 2,425 |
| #5 | "high flow therapy"[Title/Abstract] | 142 |
| #6 | "high flow oxygen"[Title/Abstract] | 890 |
| #7 | "nasal high flow"[Title/Abstract] | 267 |
| #8 | #1 OR #2 OR #3 OR #4 OR #5 OR #6 OR #7 | 3,947 |
| #9 | obesity[MeSH Terms] | 247,329 |
| #10 | "obes*"[Title/Abstract] | 361,510 |
| #11 | bariatric[Title/Abstract] | 24,837 |
| #12 | fat[Title/Abstract] | 300,199 |
| #13 | corpulent[Title/Abstract] | 217 |
| #14 | #9 OR #10 OR #11 OR #12 OR #13 | 642,523 |
| #15 | trial[Title/Abstract] | 719,496 |
| #16 | #8 AND #14 | 121 |
| #17 | #15 AND #16 | 30 |

**Source: Web of science**

**Searched on:** August 10, 2022

**Results: 66**

| Search | Query | Results |
| --- | --- | --- |
| #1 | TS=("HFNC") | 951 |
| #2 | TS=("HFNO") | 144 |
| #3 | TS=("NHF") | 635 |
| #4 | TS=("high flow nasal") | 2,643 |
| #5 | TS=("high flow therapy") | 197 |
| #6 | TS=("high flow oxygen") | 945 |
| #7 | TS=("nasal high flow") | 379 |
| #8 | #1 OR #2 OR #3 OR #4 OR #5 OR #6 OR #7 | 4,337 |
| #9 | TS=("obes*") | 492,380 |
| #10 | TS=(bariatric) | 37,390 |
| #11 | TS=(fat) | 431,720 |
| #12 | TS=(corpulent) | 374 |
| #13 | #9 OR #10 OR #11 OR #12 | 830,435 |
| #14 | TS=(trial) | 1,804,943 |
| #15 | #8 AND #13 | 142 |
| #16 | #14 AND #15 | 66 |

**Source: Embase**

**Searched on:** August 10, 2022

**Results: 59**

| Search | Query | Results |
| --- | --- | --- |
| #1 | 'high flow nasal cannula therapy'/exp | 4,005 |
| #2 | hfnc:ab,ti | 1,846 |
| #3 | hfno:ab,ti | 284 |
| #4 | nhf:ab,ti | 952 |
| #5 | 'high flow nasal':ab,ti | 4,158 |
| #6 | 'high flow therapy':ab,ti | 237 |
| #7 | 'high flow oxygen':ab,ti | 1,642 |
| #8 | 'nasal high flow':ab,ti | 446 |
| #9 | #1 OR #2 OR #3 OR #4 OR #5 OR #6 OR #7 OR #8 | 8,612 |
| #10 | 'obesity'/exp | 616,002 |
| #11 | 'obes*':ab,ti | 521,481 |
| #12 | bariatric:ab,ti | 43,096 |
| #13 | fat:ab,ti | 398,211 |
| #14 | corpulent:ab,ti | 264 |
| #15 | #10 OR #11 OR #12 OR #13 OR #14 | 1,011,701 |
| #16 | 'trial':ab,ti | 1,032,464 |
| #17 | #9 AND #15 | 455 |
| #18 | #16 AND #17 | 59 |

**Source: Cochrane library**

**Searched on:** August 10, 2022

**Results: 26**

| Search | Query | Results |
| --- | --- | --- |
| #1 | ("HFNC"):ti,ab,kw | 614 |
| #2 | ("HFNO"):ti,ab,kw | 93 |
| #3 | ("NHF"):ti,ab,kw | 169 |
| #4 | ("high flow nasal"):ti,ab,kw | 1,273 |
| #5 | ("high flow therapy"):ti,ab,kw | 109 |
| #6 | ("high flow oxygen"):ti,ab,kw | 562 |
| #7 | ("nasal high flow"):ti,ab,kw | 261 |
| #8 | #1 OR #2 OR #3 OR #4 OR #5 OR #6 OR #7 | 2,012 |
| #9 | MeSH descriptor: [Obesity] explode all trees | 15,961 |
| #10 | ("obes*"):ti,ab,kw | 90 |
| #11 | ("bariatric"):ti,ab,kw | 3,041 |
| #12 | ("fat"):ti,ab,kw | 37,408 |
| #13 | ("corpulent"):ti,ab,kw | 0 |
| #14 | #9 OR #10 OR #11 OR #12 OR #13 | 51,183 |
| #15 | (trial):ti,ab,kw | 932,902 |
| #16 | #8 AND #14 | 42 |
| #17 | #15 AND #16 | 26 |

**Source:** Google scholar

**Searched on:** August 10, 2022

**Results: 2** （searched by title from other articles）
